# Precise Spatial and Temporal Control of Oxygen within *In Vitro* Brain Slices via Microfluidic Gas Channels

**DOI:** 10.1371/journal.pone.0043309

**Published:** 2012-08-14

**Authors:** Gerardo Mauleon, Christopher P. Fall, David T. Eddington

**Affiliations:** 1 Department of Bioengineering, University of Illinois at Chicago, Chicago, Illinois, United States of America; 2 Department of Computer Science, Georgetown University, Georgetown, Washington, D. C., United States of America; University of Antwerp, Belgium

## Abstract

The acute brain slice preparation is an excellent model for studying the details of how neurons and neuronal tissue respond to a variety of different physiological conditions. But open slice chambers ideal for electrophysiological and imaging access have not allowed the precise spatiotemporal control of oxygen in a way that might realistically model stroke conditions. To address this problem, we have developed a microfluidic add-on to a commercially available perfusion chamber that diffuses oxygen throughout a thin membrane and directly to the brain slice. A microchannel enables rapid and efficient control of oxygen and can be modified to allow different regions of the slice to experience different oxygen conditions. Using this novel device, we show that we can obtain a stable and homogeneous oxygen environment throughout the brain slice and rapidly alter the oxygen tension in a hippocampal slice. We also show that we can impose different oxygen tensions on different regions of the slice preparation and measure two independent responses, which is not easily obtainable with current techniques.

## Introduction

How neuronal tissue responds at the microscale to a hypoxic insult is a fundamental question for stroke research. The hippocampal acute brain slice preparation, with its defined cytoarchitecture, mechanical stability, and recognized sensitivity to oxygen variations [1], [2], [3], provides an *in vitro* model where the effect of oxygen deprivation on neuronal physiology can be studied in isolated detail. The ability to precisely control the spatiotemporal oxygen environment in a brain slice will give us better insight into the relationship between oxygen and neuronal function in the living brain.

Most studies that subject isolated neuronal tissue to a hypoxic insult rely on perfusion chambers in which the oxygen supply is carefully regulated by the investigator [4], [5], [6], [7]. The basic techniques used to supply oxygen to the slices have changed little since their conception [8]. Recording chambers can be divided in two main groups: interface-type and submerged-type chambers. In interface-type chambers, slices are placed on a nylon mesh at the interface between artificial cerebral spinal fluid (aCSF, saturated with 95% O_2_/5% CO_2_) below the mesh, and a humidified gas mixture (usually 95% O_2_/5% CO_2_) above the mesh [9]. In submerged-type chambers, the slice is placed in the chamber and completely submerged by perfusing aCSF (saturated with 95% O_2_/5% CO_2_). In order to expose the slice to a hypoxic environment, the humidified gas mixture (for interface-type) or the oxygenated aCSF (for submerged-type) are switched to a nitrogen saturated (95% N_2_/5% CO_2_) medium and in some cases sodium cyanide is applied to a small area of the tissue with the use of a pipette [10], [11].

While the interface-type chamber provides several advantages over the submerged-type chamber, such as improved physiological network activity and rapid changes in oxygenation while maintaining mechanical stability [7], it also has several drawbacks that can be addressed by using submerged-type chambers. Due to the low flow rate used in interface-type chambers, rapid changes of pharmacological agents dissolved in the liquid media are difficult. Also, water-immersion objectives are not compatible with interface-type set-ups, which eliminates the possibility of performing visually guided patch-clamp recordings or detailed fluorescent imaging [7].

Just like its interface companion, the submerged-type chamber also has some disadvantages. Perfusion-driven oxygen delivery is not controlled enough to oxygenate the slice homogeneously; oxygen gradients form throughout the slice with the core of the slice being hypoxic compared to the edges [8], [12], [13]. Moreover, the delivery of oxygen to the brain slice cannot be precisely controlled and is cumbersome to isolate from any experimental chemicals that may be dissolved in the aCSF. Importantly, perfusion under typical protocols is all or nothing. It is impossible to selectively control oxygen levels on a scale that is spatially and temporally relevant to *in vivo* ischemia.

Microfluidic technology has provided neuroscience with tools necessary to perform powerful yet elegant brain slice experiments. In previous studies, modified perfusion chambers have been used to control the spatio-temporal delivery of chemical stimuli [14], [15], [16]. In addition, microfluidic gas channels can be used to rapidly cycle oxygen in adjacent fluidic chambers [17]. In the present study, we designed a microfluidic substrate for standard off the shelf perfusion chambers that diffuses oxygen throughout a thin membrane and directly to the brain slice. Microchannels are responsible for the rapid and efficient oxygen delivery and can be modified to allow different regions of the slice to experience different oxygen environments.

Using this device, the brain slice is in direct contact with the oxygen-permeable membrane substrate with oxygen gas channels beneath the membrane. This allows us to leverage rapid microscale diffusion to achieve a more stable and uniform oxygen environment throughout the brain slice than is possible with only perfusion. Finally, using an iteration of the diffusion device with adjacent independent microchannels, we show that we can independently oxygenate different regions of the hippocampus and measure two independent responses – thus demonstrating the utility to stroke research and neuroscience in general. It is important to remember that since we are modifying a commercially available open bath perfusion chamber, this technology can be used alongside standard electrophysiology tools.

## Materials and Methods

### Design of oxygen delivery add-on

The microfluidic channel and the membrane were fabricated out of the elastomer polydimethylsiloxane (PDMS) using soft lithography as previously described [18], [19]. Alignment marks were used to create holes in the PDMS membrane and in the perfusion chamber in such a way that they allowed the oxygen to flow into and out of the microfluidic channel below the gas permeable PDMS membrane. Once the individual parts were aligned, they were irreversibly bonded to complete the device. The oxygen gas is supplied at a rate of 38ccm to the microfluidic gas channel in order to allow adequate perfusion through the microfluidic network without distending the membrane. The perfusion chamber allows the slice to be completely submerged under the aCSF while the PDMS membrane provides a mechanically stable surface for the tissue. The optically transparent PDMS allows clear visualization of the neurons from the bottom of the device, which is necessary to measure changes in fluorescence intensity observed with the calcium indicator using an inverted microscope. Even better results should be expected when using immersion objectives and upright microscopes typically used for electrophysiology studies on slice preparations. Also, since we are modifying an open bath perfusion chamber, the top of the device allows the use of all applicable neuroscience tools associated with open bath setups.

### Fabrication of oxygen delivery add-on

The oxygen delivery add-on consists of 2 polymeric parts: the microfluidic network and the gas-permeable membrane. The photomask design was designed in Adobe Illustrator CS4 and printed on high-resolution (16,000dpi) transparency film (Fineline Imaging, Colorado Springs, CO). The design consisted of either a single or multiple large gas chambers with 500 µm cylindrical support pillars to prevent the membrane from distending or collapsing. To fabricate the negative mold master, a 3in silicon wafer was thoroughly cleaned before being exposed to oxygen plasma (Plasmatic Systems, Inc. Plasma-Preen II-862, North Brunswick, NJ). Next, the treated wafer was spin-coated (Laurel) with SU-8 2100 photoresist (MicroChem Corporation, MA) to achieve a thickness of 200 µm (spun at 1500 rpm for 30 sec). This wafer/master is then soft baked (95°C for 40 minutes), selectively exposed (315mJ/cm^2^), post-exposure baked (95°C for 14 minutes), and developed. To fabricate the positive mold of the device, 2 batches of 5 grams of polydimethylsiloxane (PDMS) (Sylgard 184 kit, Dow Corning) were prepared (10∶1 mixture of prepolymer and curing agent; degassed under vacuum). First, one of the PDMS batches was spin-coated on the master to achieve a thickness of 100 µm (spun at 800 rpm for 30 sec) followed by curing for 15 minutes at 75°C. Then, the second batch of PDMS is spin-coated on top of the master at the same speed followed by curing for 2 hours at 75°C; this combined process creates a uniform 200 µm thick PDMS layer. Once the PDMS layer is cured, it can be separated from the master mold and bonded to one 22×40 mm coverglass using oxygen plasma exposure (Plasmatic Systems, Inc. Plasma-Preen II-862, North Brunswick, NJ).

In order to make the gas-permeable membrane, 5 grams of PDMS was mixed as mentioned above. Next, the PDMS was spin-coated on a new silicon wafer to achieve a thickness of 100 µm followed by curing for 2 hours at 75°C. Once the PDMS layer was cured, a section that would fit the microfluidic network was removed from the wafer and placed on a transparency film. Following this step, using alignment marks, the inlet and outlet ports were made in the membrane using a blunted punch hole. Once the gas-permeable membrane and the microfluidic network were ready, they were exposed to oxygen plasma and bonded together, making sure that the holes in the membrane would make contact with the inlet and outlet of the network device.

### Standard perfusion chamber attachment

This process is similar to what has been previously described [16]. Briefly, inlet and outlet ports were drilled in opposite sides of the standard perfusion chamber (RC-26GPL, Warner Instruments) making sure that the ports would properly align with the oxygen delivery device. To bond the two pieces of the device, a light coating of PDMS was applied to the bottom of the perfusion chamber as an adhesive.

### Validation of device using hand-held optical sensor

A hand-held optical sensor (Neofox, Ocean optics) was used to determine the oxygen concentration inside the brain slice. The oxygen probe uses photoluminescence-quenching of a ruthenium compound to detect oxygen molecules and has a reaction time of less than 30 seconds in liquid. The sensor was calibrated according to the manufacturer's instructions, namely, 95% N_2_/5% CO_2_ and 95% O_2_/5% CO_2_ was used to represent 0% O_2_ and 95% O_2_ respectively as CO_2_ can also alter the fluorescence of the probe. The oxygen concentration inside the brain slice – the hippocampal CA1 area – was gathered in 3 different steps. First, 2 flasks with aCSF solution were bubbled with 95% N_2_/5% CO_2_ and 95% O_2_/5% CO_2_ until the aCSF's oxygen concentrations was 3±2% O_2_ and 91±2% O_2_ as measured with the optical sensor. Next, while in a standard perfusion chamber, the oxygen concentration inside the brain slice was measured while cyclic oxygenated and deoxygenated flows were applied– all experiments involving perfusion were done at a rate of 2 ml/min unless otherwise indicated. Following that test, a brain slice was placed inside the finalized oxygen delivery device. Then, the chamber was filled with aCSF (no flow for this experiment), and the oxygen concentration inside the brain slice was measured while different oxygen gasses (0%, 95% O_2_) were injected through the device. As a third test, we measured the oxygen concentration inside the brain slice while the gas was injected through the microchannels and aCSF was perfusing through the chamber (combination of oxygenation methods).

In order to measure the oxygen concentrations inside the brain slice, the oxygen probe was attached to an electronic manipulator that could maneuver the probe in the x, y, and z planes with a resolution of 0.1 µm. For our purposes, the oxygen concentration at a height (starting from the bottom of the chamber) of 0, 100, 200, 300, and 350 µm (top of the slice) were measured. We also measured the oxygen concentrations of the aCSF during the experiments.

### Brain slice preparation

The Animal Care and Use Committee at the University of Illinois Chicago approved all of the procedures outlined here. Post-natal 24 days wild type BL7 mice were deeply anesthetized using Aerrane (isoflurane, USP) and decapitated. Brains were rapidly removed from the skull and placed in chilled (3–7°C) high-sucrose cutting solution. Then, the cerebellum was separated and disposed, while the rest of the brain tissue was glued to an agar block using superglue with the cerebral cortex facing down. Next, while in high-sucrose cutting solution, 350 µm thick hippocampal slices were cut with a tissue slicer (Vibratome Series 1000 Classic) along the horizontal plane. The slices were then placed in a custom-made holding chamber containing high-sucrose cutting solution and incubated at 34°C for 35 minutes. Then, the slices were transferred to another chamber containing artificial cerebral spinal fluid (aCSF) and incubated at the same temperature for 25 minutes. Following the incubation period, the brain slices were kept at room temperature. 95% O_2_/5% CO_2_ gas was continually bubbled into all solutions the brain slices were kept in. The cutting solution contained (in mM) 82.70 NaCl, 23.81 NaHCO_3_, 2.41 KCl, 2.65 Na_2_HPO_4_, 14.53 MgCl_2_, 0.64 CaCl_2_, 23.70 Glucose and 71.19 Sucrose. The aCSF solution used during slice incubation and experiments contained (in mM): 124.98 NaCl, 23.01 NaHCO_3_, 2.50 KCl, 2.36 Na_2_HPO_4_, 0.43 MgCl_2_, 0.26 CaCl_2_, and 25 Glucose. The osmolarity of the solution was 300–310mOsm, adjusted with sucrose. All experiments were performed at room temperature.

### Validation of device via intracellular calcium response

In order to determine the intracellular calcium response of the brain slice, Fura-2/AM (acetoxymethyl ester) (Biotium) was used. A modified version of Beierlein et al. [20] Fura-2 loading protocol was used to prepare the brain slices for imaging. After finishing the aCSF incubation period, the hippocampal brain slices were stained with Fura-2/AM and incubated at room temperature for 60 minutes before imaging. Due to the long incubation period, a customized microfluidic device was used to oxygenate the brain slices with (21%O_2_/5%CO_2_) [21] which was found to enhance cellular uptake of dye. Images used to measure the calcium response were obtained from the CA1 area of the hippocampus by measuring the Fura-2 fluorescence emission at 510 nm using a fluorescent inverted microscope (Olympus IX71). The ratiometric data was obtained by exciting the samples with 340/380 nm wavelengths using the image acquisition and analysis software MetaFluor Imaging System (Universal Imaging Corp.). For statistical analysis, the ratiometric data (340 nm intensity divided by 380 nm intensity) were converted to percent change in fluorescence by dividing the ratios obtained from each image by the average intensity ratio during the baseline-recording period (initial 5 minute period) and multiplying the result by 100; the pictures were acquired using the 10X objective. The procedure used to validate the device using the optical sensor was replicated here.

### Multiple oxygen conditions on the same slice

We fabricated a second microfluidic device in order to provide not only temporal control, but spatial control as well. This second device is essentially identical to the previous device except that instead of one microfluidic channel supplying the oxygen to the entire brain slice, several independent microchannels can supply different regions of the slice with different oxygen concentrations. As with the previous device, this device docks with a common perfusion chamber (Warner Instruments, RC-26GPL). The walls dividing each individual channel are 0.3 mm wide and allow complete oxygen independence from one channel to the next. We designed this device to allow the hippocampal region to be divided in 2 sections: the CA1 area can be placed on top of one channel, while the dentate gyrus can be placed on a separate channel. We validated this concept in much the same way that we validated our first microfluidic device.

In order to simulate a stroke, we placed one of the brain slices in the device, in such a way that the dentate gyrus was on top of one of the channels, while the CA1 area was on top of a separate area. Then, we imaged the CA1 and the dentate gyrus. During this experiment, the CA1 area was exposed to different oxygen concentrations (0%, 95% O_2_), while the rest of the slice was experiencing a constant oxygen environment (95% O_2_). In a similar experiment, the Fura response, as well as the oxygen concentration, was measured at multiple positions across the channels. Using this information, we determined the spatial resolution of the microfluidic device.

### Statistical analysis

Experiments involving animal tissue were performed on a minimum of 3 brain slices obtained from 3 different animals for a total of 9 individual data sets. Experiments not involving animal tissues were repeated a minimum of three times. Graphs show the average value with the error bars representing standard deviation. In addition, we used a two-tailed, unpaired t-test (Excel) to compare the Fura imaging results obtained from the diffusion device and the perfusion method.

## Results

### Characterization of the microfluidic add-on

**Figure 1:**
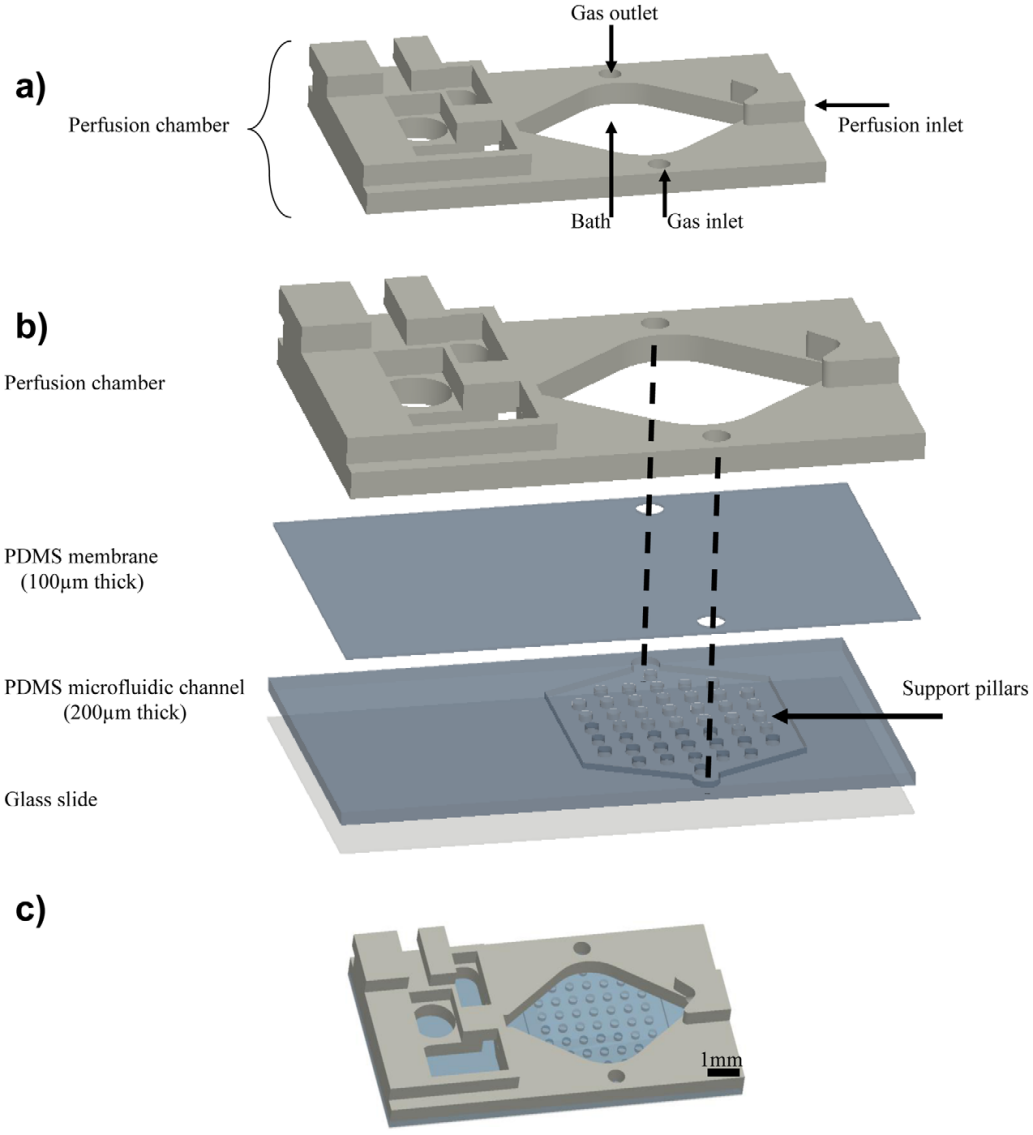
Schematic of the microfluidic device. **A.** Standard perfusion chamber (RC-26GLP). Two holes were drilled at predetermined positions in order to act as the gas inlet and outlet. **B.** Exploded view of the microfluidic device. As seen from the diagram, the device consists of 4 independent parts: the perfusion chamber, the PDMS membrane, the PDMS microfluidic channel, and a glass slide. Alignment marks show how the gas is supplied to the device. Support pillars prevent the PDMS membrane from collapsing and blocking the microfluidic channel. **C.** Picture showing the completed device.

**Figure 2:**
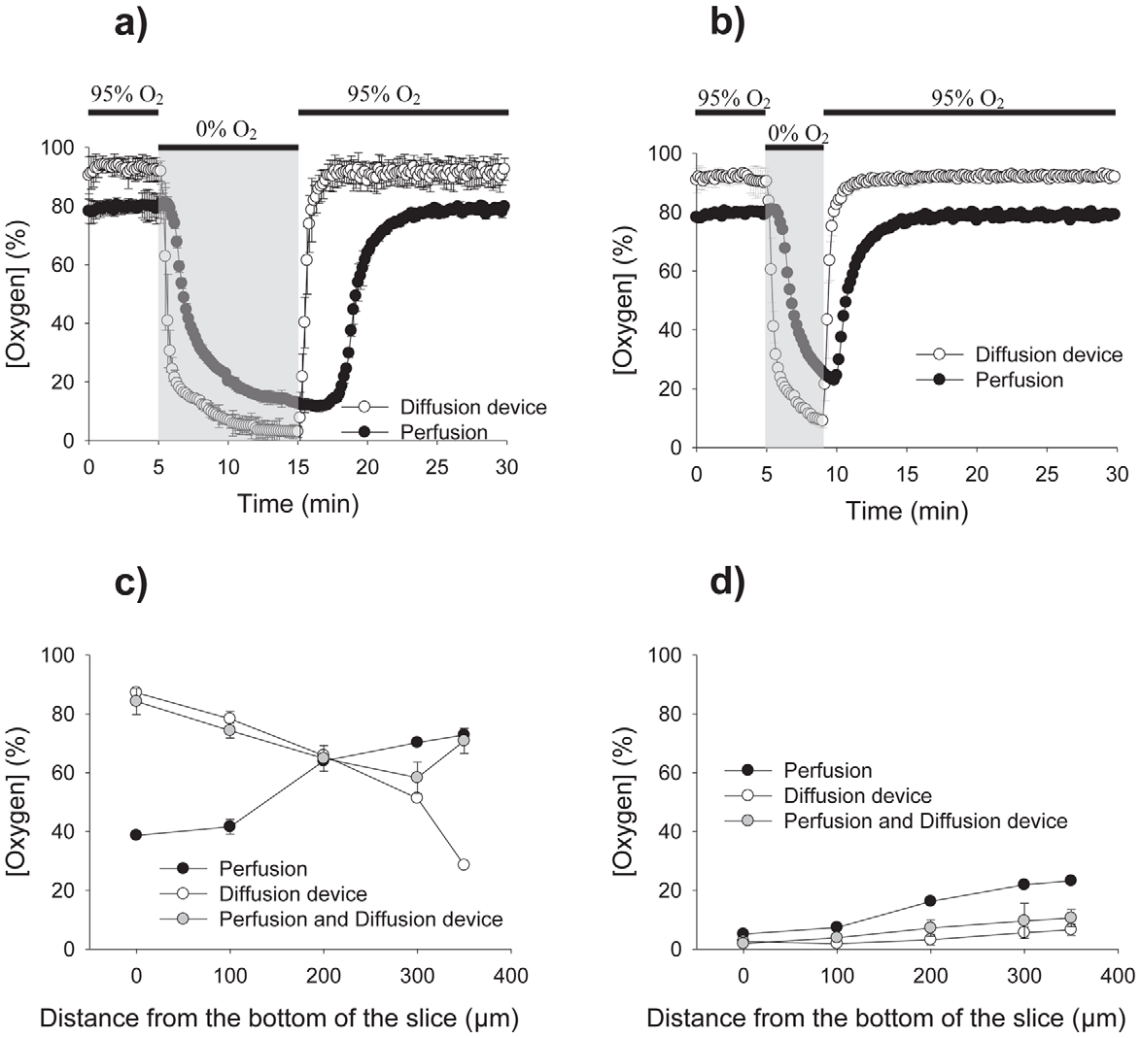
Validation of the microfluidic device with an oxygen sensor. **A–B.** Using an oxygen probe (Neofox, Ocean Optics) and a transient hypoxic stimulus lasting 10 and 4 minutes, the oxygen concentration dissolved in the aCSF was measured for the diffusion device and the traditional perfusion. No slice was present during these experiments. The diffusion device obtains a more controlled as well as a bigger change in oxygen concentration. **C.** Using the oxygen sensor mounted to an electric translation manipulator, the oxygenation at depths of 0, 100, 200, 300, and 350 µm were measured, with 0 µm marking the bottom of the chamber. Perfusion and device measurements are recorded. The graph shows the oxygen concentration during the oxygenation period (95% O_2_). **D.** The graph shows the oxygen concentration inside the tissue obtained at the end of the hypoxic stimulus.

In order to expose the brain tissue to a better controlled hypoxic environment, we developed a microfluidic add-on to a commercially available perfusion chamber (Warner Instruments, RC-26GPL) (**Fig.** **1A**). The add-on consists of 4 independent parts (**Fig.** **1B**): a glass slide for support, a microfluidic channel that delivers the oxygen (250 µm thick), a thin membrane that allows oxygen diffusion (100 µm thick), and the perfusion chamber. Even though the assembled microfluidic add-on (**Fig.** **1C**) and the perfusion method are both capable of delivering a supply of oxygen to the brain slice (**Fig.** **2A**), the device allows more complete temporal control over a hypoxic insult to the tissue and is able to reach greater differences in oxygen concentration when compared to the perfusion method. In the literature, the time used for the hypoxic insult is not consistent from study to study, but as expected, a longer hypoxic period creates a greater deoxygenation.

The device is capable of creating a hypoxic environment in less than four minutes which is faster than the previously published time of 10 minutes for the perfusion method [22], and is able to revert back to its initial settings in the same amount of time, four minutes, compared to perfusion, which requires over eight minutes to equilibrate at a fluid flow rate that is compatible with electrophysiology. The device is capable of reaching a level of hypoxia of 2% oxygen after an insult lasting 10 minutes while the perfusion method can only achieve 12% oxygen. However, one of the objectives of this study is to deliver a hypoxic stimulus in a time scale relevant to biological conditions. Considering how a hypoxic stimulus as short as 5 minutes can produce lasting damage to neuronal cells [23], we decided to use a hypoxic stimulus lasting 4 minutes for the rest of the experiments. Using this time scale, the device is also capable of achieving a level of hypoxia of 9% as compared to the perfusion method, which was only able to achieve 22% (**Fig.** **2B**).

By eliminating the need for perfusion-driven oxygenation/deoxygenation, some of the problems inherent to this method can be avoided. Switching between fluids can lead to bubble formation, pulsations in the fluid level in the perfusion chamber (using both peristaltic pumps and gravity drip feed), and depending on the flow rate, a shear stress experienced by the tissue that can lead to mechanical instability [18]. Some of this problems can be avoided if a slower flow rate is used, however this would lead to a bigger lag in the time response than is already seen when switching between fluids (**Fig.** **2A**) due to the dead volume in the slice chamber.

### Constant oxygen environment

Common methods use only perfusion to oxygenate a brain slice [24]. Because one side of the slice faces the glass bottom of the chamber, the end result is lower oxygen condition in the middle of the slice when compared to the outer edges of the slice [8], [12], [13]. Some newer methods modify the perfusion chamber in such a way as to elevate the slice in an attempt to have fluid flowing above and below the slice, however, even with this modification, an oxygen gradient within the slice is still created [25]. By measuring the oxygen concentrations inside the brain slice at various depths during the oxygenation period (**Fig.** **2C**), we demonstrated that our device created a noticeable gradient as the diffusion distance increased. This gradient moved in the opposite direction as the gradient produced by the perfusion method. By using the perfusion method and the device, a relatively uniform physiological oxygen environment is created in the brain slice. In Fig. 2D, the oxygen concentrations at the end of the hypoxic stimulus are recorded. Even though the device does not create a uniform physiological oxygen environment during the oxygenation period without the help from fluid perfusion, it is capable of producing a uniform hypoxic environment throughout the entire slice with only an 8% difference from the bottom to the top of the slice, while perfusion creates a gradient of over 20% from top to bottom. These data suggest that the device is both capable and efficient at producing hypoxic insults on the brain tissue in a well-controlled manner superior to current methods.

### Fura-2 imaging of the hippocampus

Fluorescent calcium indicators have allowed neuroscientists to use calcium as a quantitative factor to relate oxygen deficiency to neuronal viability [26], [27]. We chose to measure the response using this technique because it demonstrates the spatial control of oxygen that we are able to impose on the brain slice. Using our microfluidic device to control oxygen, we imaged the calcium response in the neurons from the CA1 area of the hippocampus to determine the relationship between neuronal function and hypoxia. The hippocampus's role in memory formation and the fact that it is particularly sensitive to oxygen level changes are well documented [28], [29], [30]. Among the different areas of the hippocampus, the CA1 area is the most vulnerable to hypoxic events, followed by the dentate gyrus that also suffers neuronal damage [31]. During a hypoxic event, overactivation of glutamate receptors allow a massive amount of calcium into the cell, which leads to a cascade of events that if not resolved, ultimately leads to cell death [32], [33]. Therefore, an increase in intracellular calcium levels is one indicator of a neuron experiencing a hypoxic insult. Using the ratiometric calcium indicator Fura-2, it is possible to quantify the extent of the intracellular calcium level increase. When the Fura-2 molecule binds to calcium, the ratio (340/380) intensity increases [34].

**Figure 3:**
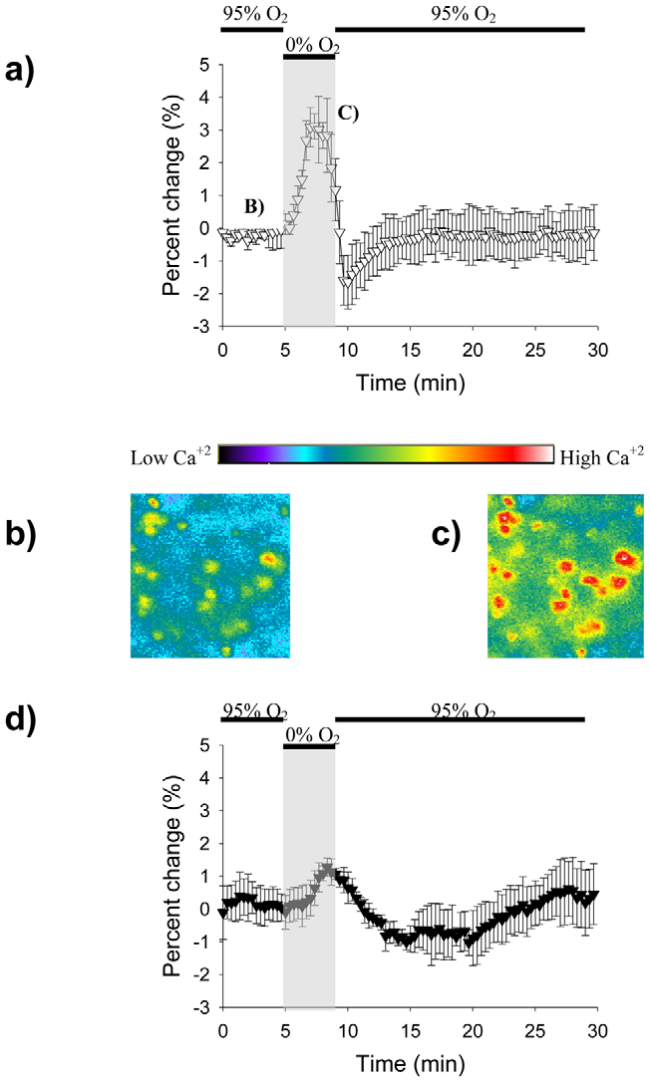
Fura readings for the diffusion device and the perfusion method. **A.** Graph showing the Fura signal obtained by the microfluidic device. Increases in signal intensity signify an increase in intracellular calcium. The hypoxic period is represented by the gray rectangle. **B–C.** 10X magnification images showing CA1 cells before and during the hypoxia period. The increase in fluorescence in C corresponds to an increase in intracellular calcium as previously mentioned. **D.** Graph showing the Fura signal obtained by the perfusion method.

To image the transient calcium levels, Fura-2/AM was bath loaded into the neuronal cells of the hippocampal area. Slices were exposed to a hypoxic insult mediated either by the microfluidic device (**Fig.** **3A**) or by the perfusion method (**Fig.** **3D**). All of the images were taken with 10X magnification and 2 sample images taken before and during the hypoxic insult are shown in Fig. 3B and 3C. Even though both methods were able to deliver the hypoxic stimuli and a response was obtained for each, the microfluidic device generates the more controlled stimuli. The device's Fura response is constrained to the four minutes in which the deoxygenation occurred, while the perfusion's Fura response extended for a period close to ten minutes, long after the deoxygenation should have stopped. Moreover, the peak intensity change response to hypoxia using our device was significantly different to what we obtained with perfusion (maximal percent change with diffusion device  = 3.09+/−0.66%, maximal percent change with perfusion  = 1.16 = /−0.29%, p<0.0001, t-test).

### Spatial control over the oxygenated region

**Figure 4:**
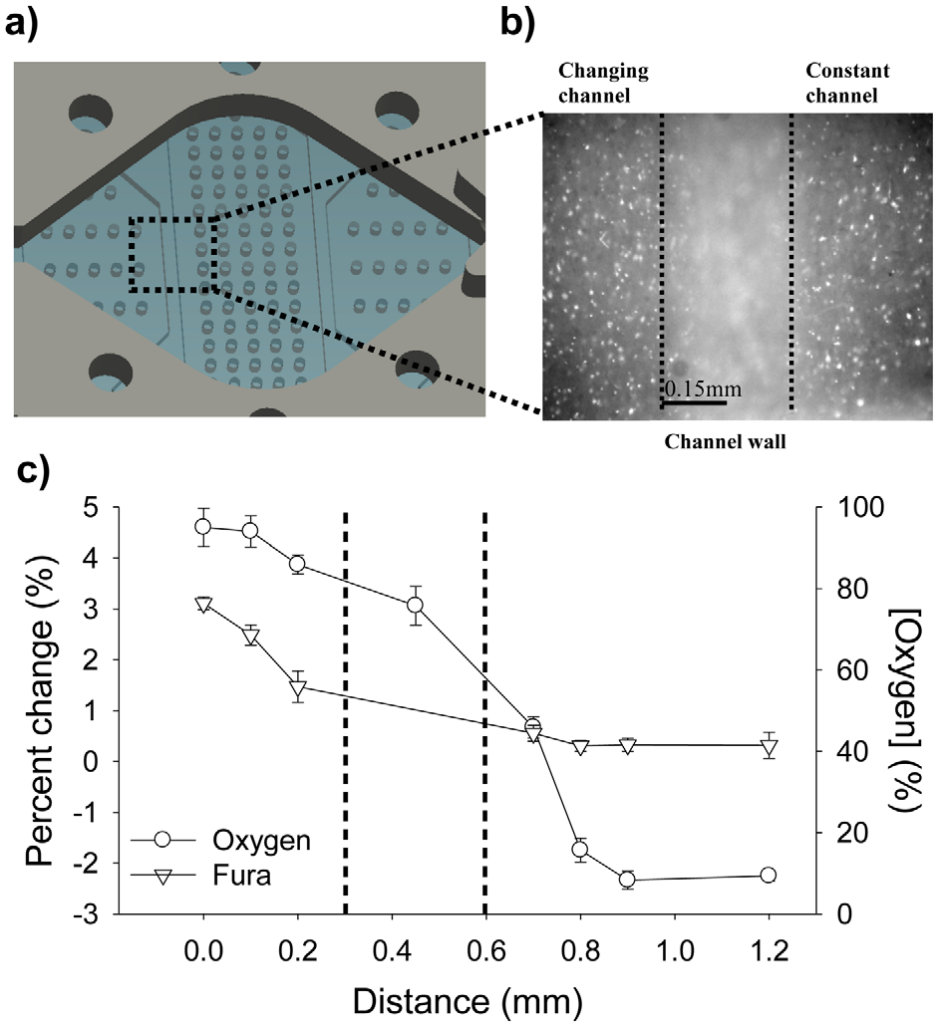
Schematic and validation of a microfluidic device capable of spatio-temporal oxygen control. **A.** Image showing a magnified view of the completed microfluidic device used to oxygenate different regions of the brain. This device consists of 4 independent parts: the perfusion chamber, the PDMS membrane, the PDMS microfluidic channels, and a simple glass slide. The square shows the location where the measurements for C were taken. **B.** 10X image showing the 2 channels used for data collection with the 0.3 mm wall at the center of the image. Data from both sides of the image were collected. **C.** To test the spatial resolution of the device, the oxygen and Fura measurements were taken at fixed positions across the microchannels. During the experiments, one channel is flowing 95% O_2_ gas, while the adjacent channel is flowing 0% O_2_ gas. As shown in the graph, there is a steep change, in both oxygen and Fura, in the measurements obtained from the 2 adjacent channels. Dashed lines indicate the wall's boundaries.

**Figure 5:**
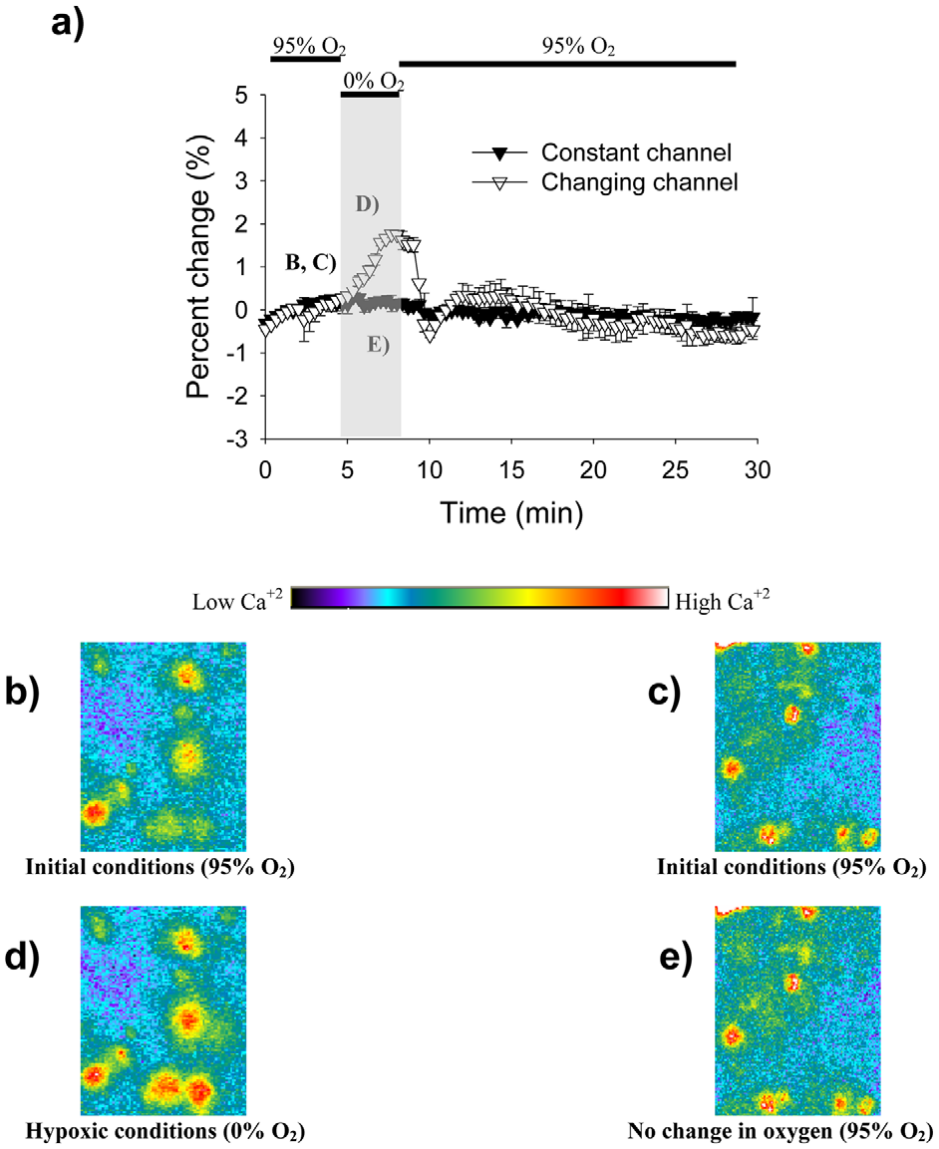
Fura readings from two different hippocampal regions. **A.** As indicated from figure 4, the microfluidic device is able to maintain 2 different oxygen concentrations in different parts of the same brain slice using microchannels. Here, the brain slice was positioned in such a way as to have the CA1 in one microchannel and the dentate gyrus in a second microchannel. The CA1 area was exposed to a hypoxic insult while the dentate gyrus was kept to a constant oxygen environment. **B, D.** These are 10X-magnification images demonstrating the increase in intracellular calcium experienced by the CA1 area. **C, E.** As expected, the dentate gyrus did not suffer a change in calcium concentration.

Another microfluidic substrate was used to allow multiple oxygen concentrations to affect different parts of the brain slice simultaneously (**Fig.** **4A, 4B**). As described previously, we validated the spatial control of oxygen levels using calcium imaging combined with direct measurement of oxygen. Figure 4C shows results when we imposed 95% oxygen and 0% oxygen in adjacent channels. The oxygen measurements show a steep change from one microfluidic channel to the adjacent one, a change mirrored by the Fura-2 calcium signal change. From the data, we can show how a distance of 100 µm away from the channel wall, in either direction, is enough to produce a different and independent calcium response. Taking in consideration the 0.3 mm wall and the 100 µm distance away from the wall, we can show that our diffusion device can spatially deliver oxygen to tissues with a resolution of 500 µm. To further illustrate spatial control of oxygenation, we recorded the Fura response of the dentate gyrus and the CA1 area while each area was exposed to a different oxygen environment (**Fig.** **5A**). In this case, CA1 was subjected to a hypoxic insult and dentate gyrus was exposed to 95% oxygen. As expected, the CA1 area showed a distinct increase in Fura intensity signifying the increase in intracellular calcium (**Fig.** **5B, 5D**), while the dentate gyrus did not exhibit any change in calcium concentration (**Fig.** **5C, 5E**). In an effort to confirm our results, we inverted the treatments with CA1 exposed to 95% oxygen and dentate gyrus subjected to a hypoxic insult. As expected, we obtained similar results (data not shown). This demonstrates the ability to deliver hypoxic stimuli with microscale precision and on time scales similar to in vivo stroke events.

## Discussion

Previously we demonstrated precise delivery of fluids including neuroactive chemicals to the acute brain slice preparation using patterned microfluidic substrates [16], [35]. But control of the neurochemical environment in acute brain slice physiology experiments implies the ability to control gases as well – most obviously oxygen. Spatiotemporal manipulation of the oxygen tension in a brain slice has not been practical using current technology. With our add-on microfluidic oxygenation device, we can adjust the spatial oxygenation conditions of subregions of the brain slice quickly and precisely using microfluidic channels and a gas permeable membrane. In our case, we chose to study the dentate gyrus and the CA1 area of the hippocampus, and thus created our device with 0.3 mm channel walls. Channel walls of this width are small enough to allow the two hippocampus' subregions to be imaged separately. At the same time, the add-on preserves the ability to maintain open access from above the chamber for electrophysiology and imaging tools. However, if smaller subregions were of interest, current microfluidic technology would make it feasible to create a microfluidic device with channel walls as small as 50 µm. If smaller walls are used, the height of the channel would need to be further reduced which would result in higher pressures in the channel due to the increased resistance and this would increase membrane deflection and possibly force gas bubbles through which would be problematic.

By manipulating oxygen to the slice both through the PDMS membrane and via the bathing aCSF, we can more homogeneously oxygenate or deoxygenate the brain slice as compared with traditional bath chambers by exposing both sides of the slice to the desired environment. The ability to more fully oxygenate the slice is an important goal, and one that has inspired several microfluidic devices [24], [36], [37]. However, based on the oxygen concentration inside the tissue that we measured, we are confident that our microfluidic device alone is capable of delivering a hypoxic insult to the cells throughout the depth of the 350 µm thick brain slice without any external perfusion. Even if measurements from the top of the slice are needed, as is the case when using electrophysiology tools, we were able to implement a physiologically relevant hypoxic insult to the tissue. Also, since the oxygen is flowing across the microchannels and diffusing throughout the PDMS membrane, manipulation of the gases does not disturb the slice mechanically as in previous efforts [3].

Our novel device has many possible applications as a physiology tool for neuroscience or for other similar tissue preparations. Along with the ability to create a more homogeneous oxygen environment throughout the brain slice, we are able to subject the brain slice to hypoxic insults at controllable rates and at defined locations within the slice. As a proof of concept, we demonstrated that the device could deoxygenate the CA1 area of the hippocampus while keeping the dentate gyrus completely unaffected. Stroke research is a prime candidate to take advantage of the ability to precisely control the spatiotemporal oxygen environment in an acute brain slice preparation. But the possibilities extend to any condition involving pathological oxygen conditions. For example it is known that in obstructive sleep apnea, intermittent hypoxia affects the hippocampus' role in learning and memory and where the CA1 and the dentate gyrus areas are affected differently [38], [39]. Furthermore, with the ability to control the oxygen environment more precisely and more easily, it might be reasonable to begin to explore the differences between *in vitro* hypoxic protocols, such as anoxia and oxygen-glucose deprivation. We have previously shown the ability to precisely control chemical delivery [16], so incorporating gas delivery (oxygen) and chemical delivery (glucose) into a single chamber is a reasonable next step.

Of course, the potential of this device is not limited to stroke research or neuroscience. Hyperoxygenation research could also take advantage of this new technology. Cyclic oxygenation is a common event throughout the body with muscle, kidney [21], and cancer cells [19] being an example. Thanks to the permeability of PDMS to gases such as hydrogen, nitrogen, helium, methane, and carbon dioxide [20], several other studies can be accomplished using this device as a way to expose different tissues to different gases.

## Conclusions

A novel microfluidic add-on to a commercially available perfusion chamber is demonstrated with the ability to spatially and temporally control the oxygen environment throughout a brain slice. Oxygen concentration recordings and ratiometric imaging experiments are performed to demonstrate that the diffusion device can oxygenate and deoxygenate the brain slice better than perfusion alone. Microchannels make it possible for the diffusion device to spatially deliver oxygen to tissues with a resolution of 500 micrometers. Even though the microfluidic add-on was demonstrated solely on brain slices, it is reasonable to expect studies using other tissues to take advantage of this technology. Ultimately, the microfluidic add-on presented here will undoubtedly lead to higher fidelity of brain slice experiments and could be generalized to any thin tissue slice preparations.
